# Good 5‐year results and a low redislocation rate using an à la carte treatment algorithm for patellofemoral instability in patients with severe trochlea dysplasia

**DOI:** 10.1002/ksa.12432

**Published:** 2024-08-22

**Authors:** Christian Dippmann, Peter Lavard, Anette Holm Kourakis, Volkert Siersma, Philip Hansen, Monica Talibi, Michael Rindom Krogsgaard

**Affiliations:** ^1^ Section of Sportstraumatology M51, Department of Orthopedic Surgery Bispebjerg and Frederiksberg Hospital Copenhagen Denmark; ^2^ Department of Public Health, The Research Unit for General Practice and Section of General Practice University of Copenhagen Copenhagen Denmark; ^3^ Department of Radiology, Copenhagen University Hospital, Bispebjerg University of Copenhagen Copenhagen Denmark

**Keywords:** Bereiter technique, patella dislocation, patella instability, trochlea dysplasia, trochleaplasty

## Abstract

**Purpose:**

Trochlear dysplasia is a major risk factor for recurrent patellar instability, reduced quality of life and osteoarthritis of the patellofemoral joint. Patellar instability in patients with trochlear dysplasia can be treated by trochleoplasty, usually in combination with medial patellofemoral ligament reconstruction (MPFL‐R). An à la carte treatment algorithm, which also addresses patella alta, lateralisation of the tibial tuberosity and valgus or torsional malalignment when present has been standard in one clinic for treatment of patellar instability patients since 2009, based on the hypothesis that it results in optimal subjective and clinical outcome, normalisation of the lateral trochlea inclination (LTI) angle and a low rate of patellar redislocation.

**Methods:**

This prospective study reports the 5‐year results for consecutive patients with high‐grade trochlea dysplasia operated according to the algorithm 2010–2017, evaluated preoperatively and 1, 2 and 5 years postoperatively. Clinical information on previous surgery and postoperative patellar stability, range‐of‐motion (ROM) and subsequent surgery were registered. Subjective outcome was evaluated by four patient‐reported outcome measures (PROMs): Kujala, Lysholm, International Knee Documentation Committee and Knee injury and Osteoarthritis Outcome Score. The LTI angle was measured pre‐ and postoperatively on magnetic resonance imaging scans.

**Results:**

There were 131 patients (87 females) with a median age of 22 years (range: 14–38). All had a trochleoplasty and an MPFL‐R. Additional procedures (tibial tuberosity medialisation/distalisation and/or femoral/tibial osteotomy) were performed in 52%. All PROM scores improved from preoperatively to 1‐year follow‐up with further improvement at 2 and 5 years after surgery (*p* < 0.05). Three patients (2%) had a traumatic patellar dislocation 9, 12 and 24 months postoperatively and 38% underwent subsequent surgery (hardware removal, arthroscopically assisted brisement force, knee arthroscopy). A normalisation of the LTI angle (≥11°) was achieved in 76%.

**Conclusions:**

Treatment according to the à la carte algorithm for patients with patellar instability and high‐grade trochlear dysplasia resulted in significant clinical and subjective improvement in all PROM scores and a very low redislocation rate (2%) 5 years after surgery.

**Level of Evidence:**

Level II.

**Abbreviations**
95% CI: 95% confidence interval
ADL: activity of daily living
ET: Elmslie–Trillat
IKDC: International Knee Documentation Committee
KOOS: Knee injury and Osteoarthritis Outcome Score
LTI: lateral trochlea inclination
MCID: minimal clinical important difference
MDE: minimal detectable effect
MPFL‐R: medial patella‐femoral ligament reconstruction
PROM: patient‐reported outcome measure
QOL: quality of life
ROM: range of motion
SD: presurgery standard deviation
TD: trochlear dysplasia
TP: trochleoplasty
TT‐TG: tibial tuberosity trochlea groove

## INTRODUCTION

Chronic patellofemoral instability typically develops during early adolescence. The risk of patellar dislocation increases with predisposing factors: patella alta, lateralisation of the tibial tuberosity, valgus/torsional malalignment and in particular trochlea dysplasia [1, 9, 10, 19, 25]. Patellar dislocation incidence varies from 42 per 100,000 in the general Danish population [15] to 148 per 100,000 among 14‐ to 18‐year‐olds in an American cohort [35]. Surgery reduces the redislocation rate to 0%–8% [40] and improves clinical function. The Dejour and the Bereiter techniques are the most commonly used trochleoplasty techniques [3, 7, 11].

The indication for trochleoplasty is widely discussed among orthopaedic surgeons [14], and the algorithms for treatment vary markedly between clinics—various radiographic indices are used as basis for the indication to perform a trochleoplasty. Lateral trochlea inclination (LTI) angle and trochlear boss height are the most common but not comparable indices, meaning that patients can be treated differently, depending on which index is preferred where they are operated. Therefore, there is a need for randomised studies and well‐defined treatment series with patients treated after strict algorithms, as they provide an indication of whether stability and subjective outcomes after the particular algorithms are satisfactory. In addition, long‐term follow‐up studies are necessary, as early results may deteriorate with time.

**Figure 1:**
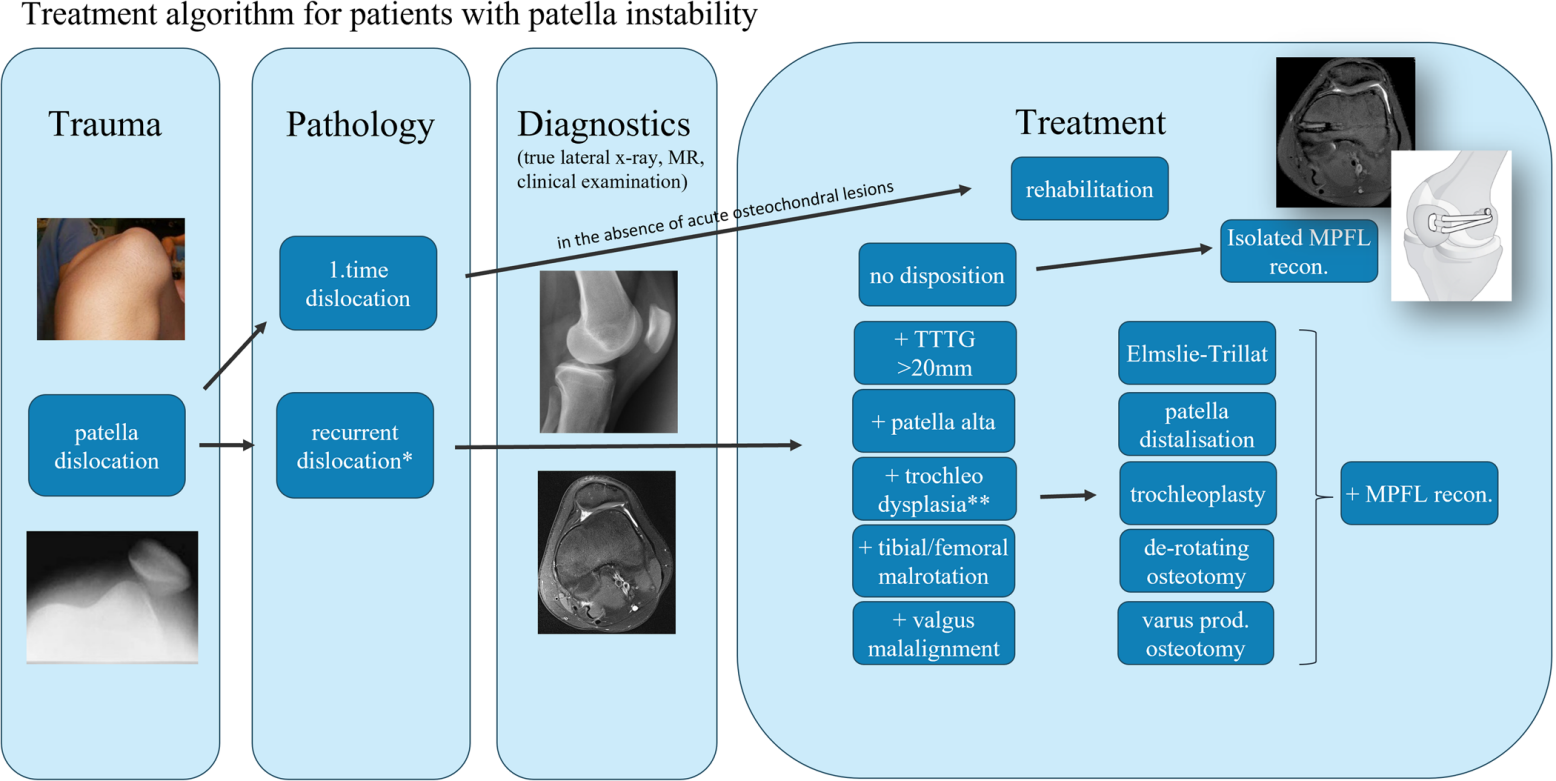
Treatment algorithm for patients with patellar instability. Patients with recurrent dislocations or permanent/habitual patellar dislocation are examined clinically and by true lateral radiograph, magnetic resonance imaging (MRI) ± computed tomography scan to identify all relevant predisposing factors. The following cut‐off values are used: tibial tuberosity trochlea groove (TT‐TG) > 20 mm on MRI scan for medialisation of the tibial tuberosity (Elmslie–Trillat), Caton‐Deschamps index > 1, 2 for distalisation of the tibial tuberosity, lateral trochlea inclination < 11° for trochleoplasty, tibial torsion >40° and/or femoral torsion >35° for derotating osteotomy and valgus angle >10° for varus‐producing osteotomy. MPFL, medial patellofemoral ligament.

As per Danish Board of Health regulations, surgical treatment for trochlear dysplasia in the Danish public healthcare system is centred at Aarhus University Hospital and Copenhagen University Hospital Bispebjerg. Since 2009, an à la carte approach to surgery has been standard at Bispebjerg Hospital. This is based on an analysis of anatomical factors of relevance for patellar instability, and all pathological factors are subsequently corrected. All patients in the current cohort were treated with trochleoplasty and medial patellofemoral ligament reconstruction (MPFL‐R), and this was combined with medialisation/distalisation of the tibial tuberosity, varus‐producing tibial/femoral osteotomy, derotational osteotomy and soft tissue procedures (lateral release/vastus plasty) when necessary for correction of all anatomical pathologies. The algorithm is described in detail in Figure 1.

This study presents the 5‐year outcome for 135 consecutive patellar instability patients who were treated with trochleoplasty as part of this à la carte treatment algorithm. The study includes 72% of all trochleoplasties performed in Denmark during this period (information retrieved from The Danish National Patient Registry on 15 July 2023).

It was hypothesised that the treatment algorithm would secure good clinical and subjective results, normalisation of the LTI angle and a low patellar redislocation rate.

By presenting a prospectively followed, consecutive cohort the current study adds substantial evidence to the pool of evidence on how these patients are optimally treated.

## MATERIALS AND METHODS

All patients gave written consent to have their data registered for the study. Data were stored electronically after permission from the Capital Region (P‐2020‐573). The ethical committee of the capital region of Copenhagen has stated that ethical permission was not necessary for this study (Journal‐nr.: H‐20031969), as the study did not involve any intervention.

According to the treatment algorithm (Figure 1), used since August 2009, first‐time patella dislocations are treated nonsurgically with a knee brace for 2 weeks and referral to structured municipal rehabilitation, unless there is a free osteochondral lesion that can be repaired. Patients with recurrent patellar dislocation, episodes of subluxation (a feeling that patella is on its way to dislocation) or lack of confidence in patellar stability are evaluated with a true lateral radiograph and a magnetic resonance imaging (MRI) scan to identify possible predisposing anatomical factors for patellar instability. Trochlear dysplasia is characterised according to Dejour [9] and LTI angle. Trochleoplasty is indicated in patients with Dejour class B or D in combination with an LTI angle <11° [9]. The LTI angle is measured by a modified two‐image technique [18]. Lateralisation of the tibial tuberosity is defined by the tibial tuberosity trochlear groove (TT‐TG) distance [36], and a pathological TT‐TG distance is defined as >20 mm on an axial MRI scan. Patella alta is defined as a Caton‐Deschamps index >1.2 [6]. Patients with patellar instability have been examined for varus/valgus as well as torsional deformity since establishment of the algorithm, and measurement of rotational alignment has been formally added to the algorithm in 2016. In cases of increased internal rotation of the hip (>45° in supine position with knee in 90° of flexion) with/without in‐toing of the feet and/or a “squinting patella sign”, rotational alignment is measured using whole leg computed tomograpphy scans, according to Waidelich et al. [40]. Derotational distal femoral osteotomy is indicated when femoral anteversion exceeds 35°, while derotating tibial osteotomy is performed when external rotation is >40°. If axial (valgus) malalignment is suspected clinically, the lower extremity long axis is evaluated using full length, standing radiographs. Varus‐producing femoral osteotomy is considered if knee valgus exceeds 10° [8].

Patients with recurrent patellar dislocations or residual patellar instability without dislocation are offered patella stabilising surgery, addressing all identifiable abnormalities (Figure 1). Trochleoplasty is performed in skeletally mature patients only. There is no upper age limit for trochleoplasty, but it is not performed in patients with grade 4 changes of femoral cartilage [8].

### Surgical technique

**Figure 2:**
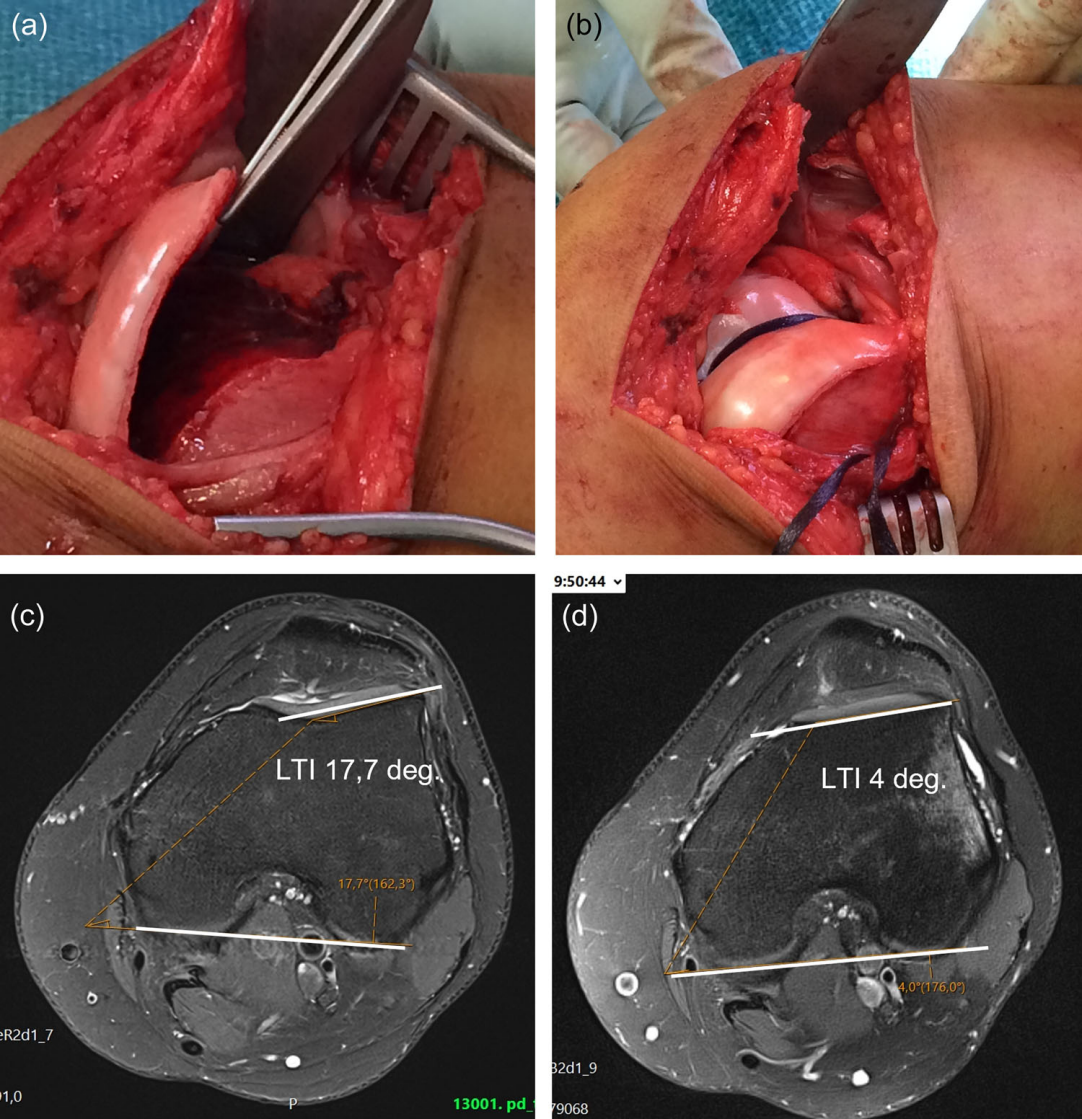
During the Bereiter trochleoplasty, a thin cartilage flap is released from the femoral trochlea (a). A new trochlea is created in the bone using chisels and a highspeed burr, keeping as much height as possible on the lateral facet of the trochlea to normalise the inclination angle. The shape of the patellar joint surface is also considered when the trochlear groove is created to secure patello‐femoral congruency. The cartilage flap is modelled into the groove and fixed centrally with a resorbable 10 mm Vicryl tape (Ethicon, Johnson & Johnson) which is taken through two drillholes to the lateral femoral condyle where it is tied (b). The increase of the lateral trochlea inclination (LTI) angle from pre‐ (d) and postoperatively (c) can be seen on an axial magnetic resonance imaging scan.

The aim of the surgical procedure was to address all morphological risk factors in patients with recurrent patellar instability as described above. The surgical technique (Figure 2) is described in the Data S1.

### Radiological measures

For measurement of the LTI angle a modified two‐image technique was used, as described by Joseph et al. [18], but using the axial image with the best definition of the posterior femoral condyles and the most proximal image with cartilage covering both facets of the trochlea (usually the third most proximal axial image on MRI with a slice thickness of 3 mm). The LTI angle was defined by osseous contours to avoid false negative LTI angles due to focal cartilage changes. The LTI angle was measured for this follow‐up study in each patient before and after surgery independently by two experienced orthopaedic surgeons and a radiologist and the measurement technique was validated by inter‐rater intraclass correlation, measured by the fraction of residual variance in the total variance assessed in a linear mixed model including a random effect for rater. Agreement was evaluated by 95% limits of agreement, calculated by the empirical 95% reference interval (2.5% and 97.5% percentiles) of the three sets of LTI angle differences between two raters (Data S2).

### Patient cohort

All patients receiving patella stabilising surgery that included a trochleoplasty from 2010 to 2017 were enroled. The cohort did not include patients who did not require trochleoplasty according to the algorithm. Sex, age, radiological data and information about previous, as well as postoperative additional surgery were registered.

### Outcomes

Patients completed four patient‐related outcome measures (PROMs): the Kujala score [20], the International Knee Documentation Committee Subjective Score (IKDC) [17], the Knee Injury and Osteoarthritis Outcome Score (KOOS) [33] and the Lysholm score [38]. Details on the PROMs are given in Data S3. Follow‐up MRI was performed at one or several of the visits 1, 2 and 5 years postoperatively and patients were asked to complete PROMs at each follow‐up visit. Patients who had not attended the 5‐year follow‐up were asked by telephone or mail whether patella was stable and whether they had experienced redislocation after the operation. The postoperative LTI was measured on the 1‐year postoperative MRI scans.

### Statistical analysis

No minimal clinically important difference has been established for any of the PROMs used in this study for patients with patellofemoral instability. Yet, a change of 50% of the preoperative standard deviation (SD) between preoperative and postoperative scores is recognised as a rough estimate of the threshold for patient‐perceived change [23]. Mean difference from baseline for the LTI and for the PROM outcomes and their 95% confidence intervals (95% CIs) were estimated in mixed linear regression models featuring a patient random effect to adjust the variance for repeated observations on the same person. These estimates were adjusted for patient age, whether there was previous surgery and whether there was a subsequent surgery after trochleoplasty.

Subgroup analyses were also performed in the mixed linear regression models described above, where an interaction between follow‐up time and the binary subgroup variable was added. Analyses were performed in SAS PROC MIXED, SAS version 9.4.

Notably in the subgroup analyses, the samples may be too small to demonstrate relevant change (i.e., a change in score larger than 0.5 SD). Therefore, for each subgroup, we calculated the change that can be detected with the available sample size; if this is higher than the change that is considered relevant, the particular analysis is underpowered. This minimal detectable effect (MDE) with power 80% (0.8 MDE), that is, the size of the change that can be found with 80% probability with 5% statistical significance with the available data, was calculated by multiplying half the width of the corresponding 95% CI with a factor ϕ = 1.43, see Liu [22]. Hence, if for an analysis 0.8 MDE > 0.5 SD this is indicative of insufficient power.

## RESULTS

During the study period, 135 knees in 131 patients (87 females, 44 males) with an average age of 22 years (range: 14–38) were treated with a trochleoplasty according to the à la carte algorithm. 90% attended the follow‐up consultations and had at least one postoperative MRI scan, while 76% of the patients completed the 5‐year PROMs (Table 1). In 43% of the cases, the patients only received the basic treatment consisting of trochleoplasty combined with an MPFL‐R. This was supplemented with one additional procedure in 35%, with two additional procedures in 20% of the cases and in 2% of the cases with more than two. The most common additional procedures were medialisation of the tibial tuberosity (in 31%) and medialisation plus distalisation of the tibial tuberosity (in 19%). Table 1 provides an overview of all additional procedures.

**Table 1:** Characteristics for the 131 patients in the cohort, including information on previous surgery, subsequent surgery, complications and completeness of the follow‐up.

Patient characteristics	
Patients	131 (135 knees)
Side	
Left	68
Right	67
Bilateral	4
Age (year)	
22	[18–38]
Sex	
Female	87
Male	44
Previous surgeries	47 (35%)
MPFL‐R	9
MPFL‐R + ET	9
ET	5
Soft tissue procedure (medial reefing, etc.)	16
Others	8
Procedures performed	
Trochleoplasty	135
MPFL‐R	135
ET	42 (31%)
ET + Tibial tuberosity distalisation	25 (19%)
TT distalisation	3
Arthroscopy	3
Vastus lateralis plasty	2
Double osteotomy (tibial and femoral)	3
Subsequent surgery	
In total	51 (38%)
Arthroscopically assisted brisement forcé	25 (19%)
Hardware removal	9
Knee arthroscopy	8
Revision MPFL‐R	3
Revision MPFL + ET	1
Second look	2
Cosmetic scar revision	2
Total knee arthrosplasty	1
Complication	
Redislocation	2 (1.48%)
Major complications (deep vein thrombosis, infection, revision TP, etc.)	0
Completeness	
PROMS	
Preoperative	66%
1 year	81%
2 years	67%
5 years	79%
Follow‐up	
2 years	85%
5 years	90%

Abbreviations: ET, Elmslie–Trillat; MPFL‐R, medial patellofemoral ligament reconstruction; TP, trochleoplasty; TT, tibial tuberosity.

Previous surgery had been performed in 47 knees (35%), in most cases soft‐tissue procedures like medial reefing and MPFL‐R. Subsequent surgery was performed in 38% (51 knees), including arthroscopic release and brisement force for limited range of motion (ROM) in 19% (24 cases). In 18 of these 24 cases ROM normalised, four gained near‐normal flexion (120–140°), while two lacked follow‐up ROM data. Four cases required revision‐MPFL‐R: two for posttraumatic instability, one for recurrent feeling of instability and one for pain in knee flexion. In one case, recurrent joint effusion was treated by synovectomy and a 6‐week antibiotics course on the suspicion of infection, although microbiological cultures were negative. At 3 months, this patient had full ROM and patellofemoral joint stability. Notably, no major complications (e.g., deep vein thrombosis, confirmed deep joint infection, trochleoplasty revision) occurred. However, one patient had a total knee arthroplasty 5 years after trochleoplasty due to severe patella‐femoral joint cartilage degeneration. This 32‐year‐old patient had grade 3 cartilage lesions by the time of trochleoplasty. Three patients (2%) had a patellar redislocation, respectively 9, 12 and 24 months after surgery. Specific information about stability (no redislocation) could be obtained in all but four patients (98%).

**Figure 3:**
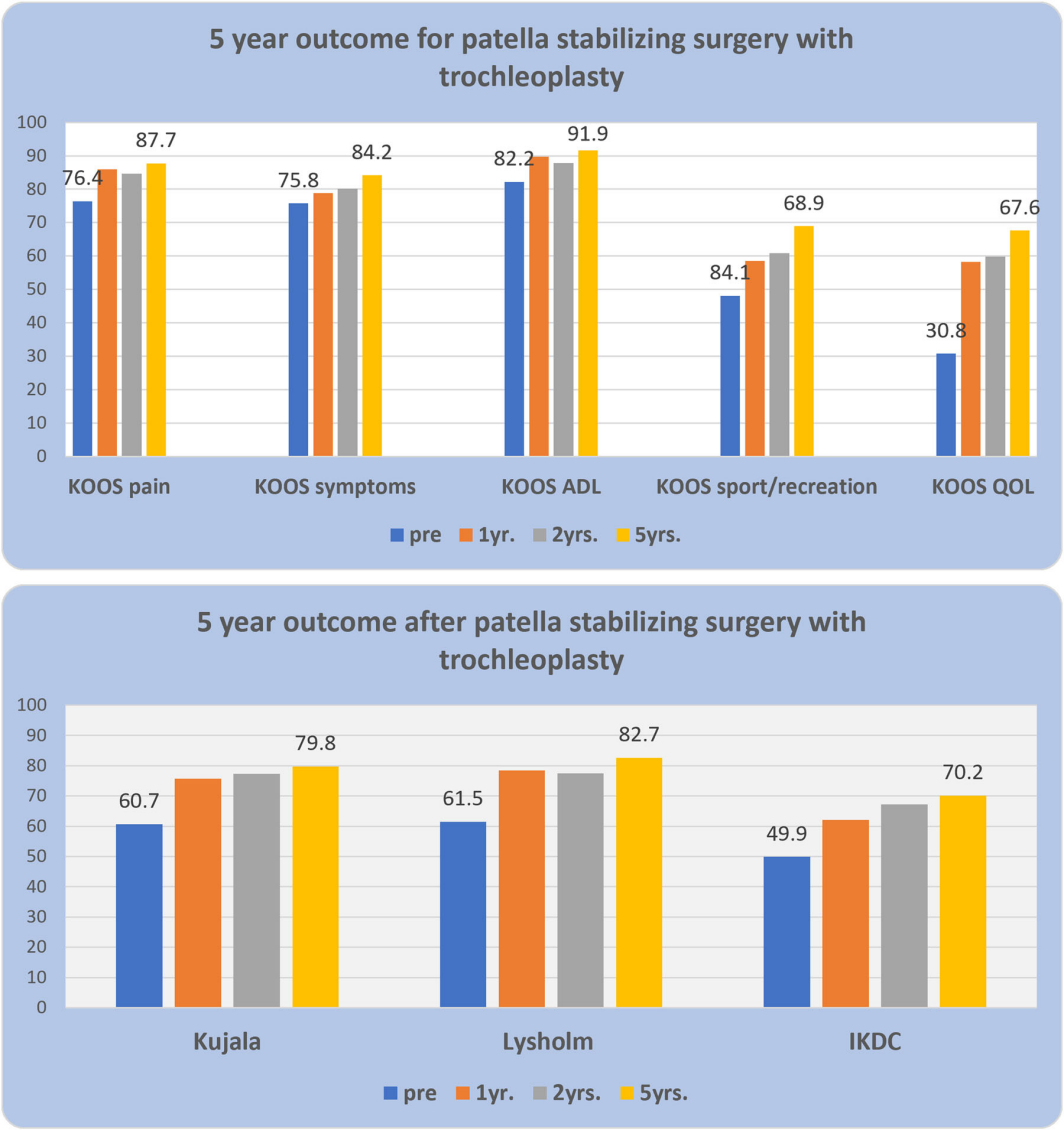
There were significant improvements in all patient‐reported outcome measure scores (above: Knee injury and Osteoarthritis Outcome Score [KOOS], below: Kujala, Lysholm, International Knee Documentation Committee [IKDC]) 1, 2 and 5 years after patella stabilising surgery according to the algorithm in patients with trochlear dysplasia. ADL, activity of daily living; QOL, quality of life.

The improvements in PROM‐scores 1, 2 and 5 years postoperatively (Figure 3, Table 2) were all statistically significant and as they were much larger than the patient‐perceived relevant changes (calculated as 0.5 SD) they can also be regarded as clinically relevant. The finding that patients who had pre‐ or postoperative surgery had lower increases in PROM scores (Table 3) was not significant (n.s.), but this may be a consequence of the reduced power caused by the smaller samples.

**Table 2:** The mean difference in PROM scores from baseline to 1,2 and 5 years after surgery, adjusted for age, treatment before and subsequent surgery for the 131 patients in the cohort.

	Baseline				1‐year follow‐up				2‐year follow‐up				5‐year follow‐up			
	*n*	Mean	Range	0.5 SD^a^	*n*	Mean	Range	Mean difference (95%CI)^b^	*n*	Mean	Range	Mean difference (95%CI)^b^	*n*	Mean	Range	Mean difference (95%CI)^b^
Kujala	70	60.7	13–92	8.3	100	75.7	33–100	14.3 (10.9; 17.8)	84	77.3	29–100	15.0 (11.5; 18.6)	99	79.8	27–100	17.8 (14.3; 21.2)
KOOS pain	66	76.4	33–100	8.5	100	86	25–100	9.2 (5.7; 12.8)	83	84.7	25–100	7.3 (3.6; 11.0)	101	87.7	31–100	10.5 (7.0; 14.0)
KOOS symptoms	66	75.8	15–100	9.1	100	78.8	32–100	3.3 (−0.4; 6.9)	82	80.2	32–100	4.4 (0.6; 8.2)	102	84.2	39–100	8.2 (4.6; 11.7)
KOOS ADL	66	82.2	25–100	8.7	100	89.7	41–100	7.9 (4.3; 11.5)	83	87.8	10–100	5.6 (1.9; 9.3)	101	91.6	28–100	9.6 (6.0; 13.1)
KOOS sport/recreation	64	48.1	0–100	14.4	99	58.5	0–100	11.2 (4.9; 17.6)	83	60.8	0–100	13.2 (6.7; 19.7)	101	68.9	0–100	21.2 (15.0; 27.5)
KOOS QOL	66	30.8	0–75	7.6	100	58.2	0–100	28.8 (24.2; 33.4)	83	59.8	0–100	30.3 (25.5; 35.0)	101	67.6	0–100	37.5 (32.9; 42.1)
Lysholm	40	61.5	31–95	9.3	76	78.4	23–100	18.1 (13.0; 23.1)	69	77.5	22–100	16.4 (11.3; 21.5)	82	82.7	26–100	21.3 (16.3; 26.2)
IKDC	58	49.9	21–89	9.0	96	62.1	23–100	12.1 (7.7; 16.5)	80	67.2	17–100	16.3 (11.8; 20.9)	96	70.2	8–100	19.6 (15.2; 24.0)

Abbreviations: ADL, activity of daily living; CI, confidence interval; IKDC, International Knee Documentation Committee; KOOS, Knee injury and Osteoarthritis Outcome Score; PROM, patient‐reported outcome measure; SD, standard deviation; QOL, quality of life.

^a^ 50% of the preoperative SD (0.5 SD) is a rough estimate for the threshold for patient‐percieved change.

^b^ Mean difference from baseline and 95% CIs. Adjusted for age, treatment before and treatment after operation.

**Table 3:** The mean difference in PROM scores from baseline to 5 years after surgery for subgroups of patients with/without previous surgery, with/without subsequent surgery and LTI > and <0°.

	No previous surgery			Previous surgery			
	*n*	Mean difference (95% CI)^a^	0.8 MDE^b^	*n*	Mean difference (95% CI)^a^	0.8 MDE^b^	*p* Value^c^
Kujala	68	18.4 (14.4; 22.4)	9.65	31	15.5 (11.4; 19.5)	5.72	n.s.
KOOS pain	69	9.8 (5.7; 14.0)	9.65	32	9.9 (5.7; 14.1)	5.93	n.s.
KOOS symptoms	70	6.6 (2.4; 10.8)	9.86	32	3.3 (−1.0; 7.6)	6.00	n.s.
KOOS ADL	69	8.8 (4.6; 13.0)	9.79	32	6.6 (2.4; 10.9)	6.00	n.s.
KOOS sport/recreation	70	19.9 (12.6; 27.2)	17.51	31	11.3 (3.9; 18.7)	10.43	n.s.
KOOS QOL	70	40.0 (34.8; 45.3)	12.58	31	31.4 (26.1; 36.8)	7.50	n.s.
Lysholm	56	22.6 (16.6; 28.6)	13.01	26	19.2 (13.1; 25.4)	8.58	n.s.
IKDC	67	20.2 (15.0; 25.4)	12.01	29	12.8 (7.5; 18.0)	7.43	n.s.

	No subsequent surgery			Subsequent surgery			
	*n*	Mean difference (95% CI)^a^	0.8 MDE^b^	*n*	Mean difference (95% CI)^a^	0.8 MDE^b^	*p* Value^c^
Kujala	70	20.0 (15.9; 24.2)	8.93	29	12.8 (6.6; 19.1)	6.51	n.s.
KOOS pain	71	11.8 (7.7; 15.9)	9.65	30	7.0 (0.2; 13.7)	5.85	n.s.
KOOS symptoms	72	9.7 (5.5; 13.9)	9.79	30	4.0 (−2.8; 10.9)	6.00	n.s.
KOOS ADL	71	10.4 (6.2; 14.6)	9.72	30	7.3 (0.5; 14.1)	6.00	n.s.
KOOS sport/recreation	71	24.2 (16.8; 31.5)	16.87	30	13.4 (1.6; 25.2)	10.51	n.s.
KOOS QOL	71	40.1 (34.8; 45.5)	12.44	30	30.4 (21.7; 39.1)	7.65	n.s.
Lysholm	59	21.8 (16.1; 27.6)	13.73	23	19.6 (10.0; 29.2)	8.22	n.s.
IKDC	67	21.0 (15.7; 26.2)	11.44	29	16.6 (8.6; 24.6)	7.50	n.s.

	LTI > 0			LTI ≤ 0			
	*n*	Mean difference (95% CI)^a^	0.8 MDE^b^	*n*	Mean difference (95% CI)^a^	0.8 MDE^b^	*p* Value^c^
Kujala	64	16.2 (11.8; 20.6)	6.29	28	21.7 (15.6; 27.8)	8.72	n.s.
KOOS pain	66	10.0 (5.6; 14.3)	6.22	29	11.4 (5.2; 17.7)	8.93	n.s.
KOOS symptoms	66	9.1 (4.7; 13.5)	6.29	29	6.7 (0.5; 13.0)	8.93	n.s.
KOOS ADL	65	8.8 (4.4; 13.2)	6.29	29	11.4 (5.0; 17.7)	9.08	n.s.
KOOS sport/recreation	65	18.6 (10.7; 26.6)	11.36	29	27.1 (16.0; 38.3)	15.94	n.s.
KOOS QOL	65	33.9 (28.3; 39.4)	7.93	29	41.6 (33.7; 49.5)	11.29	n.s.
Lysholm	53	18.7 (13.0; 24.3)	8.08	23	26.8 (17.6; 36.0)	13.15	n.s.
IKDC	61	17.8 (12.6; 23.1)	7.50	29	24.6 (17.1; 32.1)	10.72	n.s.

Abbreviations: ADL, activity of daily living; CI, confidence interval; IKDC, International Knee Documentation Committee; KOOS, Knee injury and Osteoarthritis Outcome Score; LTI, lateral trochlea inclination; MDE, minimal detectable effect; PROM, patient‐reported outcome measure; SD, standard deviation; QOL, quality of life.

^a^ Mean difference from baseline. Adjusted for age, surgery before and surgery after operation, if not superfluous.

^b^ Minimal detectable effect with 80% power.

^c^ *p* Value for difference in effect between the subgroups.

The LTI angle increased from median (range) 2.2° (−16.9 to 10.8) preoperatively to 13.7° (4–26.2) postoperatively (Table 2). A LTI angle ≥11° was achieved in 76% of all knees, in 84% of knees with a preoperative LTI angle >0° and in 44% of knees with a negative preoperative LTI angle. PROM‐scores at 5‐year follow‐up from patients with preoperative LTI angle <0° were higher than from patients with a preoperative LTI angle >0°, however, not significantly (n.s.).

### Data completeness

MRI scans were analysed for 91% preoperatively and 90% postoperatively. PROM data completeness is shown in Table 1. Information about redislocation of patella in the follow‐up period was 98%.

## DISCUSSION

The most important finding of the present study was that patients with recurrent patellofemoral instability and trochlear dysplasia had a significant long‐term clinical improvement after treatment following the standardised à la carte algorithm, by which anatomical pathologies predisposing for patellar dislocation are defined and addressed in a single procedure. Patellar dislocation is the main indication for patella‐stabilising surgery, and a 5‐year redislocation rate of 2% following the current surgery can be considered excellent. It is lower than reported after trochleoplasty in general (2.4%) and after Bereiter trochleoplasty (4%) [21, 34].

Generally, Bereiter trochleoplasty shows encouraging results [2, 12, 13, 26, 27, 28, 30, 31, 39, 41], but most studies report short‐term outcomes (<3 years) [2, 13, 28, 31, 39] and completeness of PROM data is varying with figures as low as 42% [26, 30, 39]. The current prospective study of a group of consecutive patients has a high follow‐up rate and a small risk of bias and is a substantial contribution, supporting that there is good outcome following the à la carte principle. However, the radiological thresholds for operation are frequently discussed for all elements of the á la carte algorithm [12, 14, 37]. In this study, the indication for trochleoplasty was Dejour types B and D trochlear dysplasia [8] in combination with a LTI angle <11° [29], while others prefer to use the boss height [24]. Obviously, the optimal thresholds can only be identified through randomised, controlled trials, but at the moment such studies are difficult to perform, as there is no valid PROM for this patient group [16]. This reduces the possibility to identify the most optimal treatment strategy.

Postoperative normalisation of the LTI angle (LTI ≥ 11°) was achieved in 76% of the patients. Cases with a negative LTI angle are usually more challenging surgically [29], and only 44% of patients with a preoperative LTI angle <0° had a postoperative LTI above 11°. However, as a low LTI angle is considered to be an important measure of trochlea dysplasia [14, 32], these are encouraging results.

In the current study, the LTI angle was estimated using a modified two‐image technique [18], which is considered more accurate compared to a single‐slice technique [4]. In a single‐slice technique, the LTI angle can be underestimated using an MRI slice close to the notch, while the correct femoral orientation is difficult to estimate in more proximal MRI slice [18, 42]. The method had a very high agreement between observers (98.3%) in relation to determine whether the preoperative LTI angle was <11°, which was the cut‐off value for trochleoplasty in the algorithm.

Reported rates of subsequent surgery vary from 11.1% for Bereiter trochleoplasty to 57.8% for wedge osteoplasty [21]. In the current study, 38% of patients underwent subsequent surgery, with 19% (*n* = 25) having arthroscopic release and brisement force for restricted ROM. Early intervention (within 12 weeks) in case of reduced ROM, as well as the fact that a longer observation period increases the likelihood of subsequent surgery may contribute to this relatively high rate of subsequent surgery [21]. However, only four patients (3.2%) underwent revision surgery due to recurrent instability.

Postoperative joint stiffness is a common complication following trochleoplasty [14, 21], and it may negatively influence the subjective patient outcome. Furthermore, nonoperative and operative treatment for postoperative joint stiffness is prolonging the overall recovery time. When indicated, arthroscopically assisted brisement force is usually performed within the first 12 weeks after the primary procedure, potentially exposing the patient to additional complications [21]. Wind et al. [41] reported that 46% had postoperative flexion restrictions at follow‐up after trochleoplasty, leading to reintervention in 13.6%. In a cohort of 62 trochleoplasty patients, 17.7% had arthrofibrosis with 14.5% needing surgery for this. However, the final ROM, PROM‐scores (Kujala, IKDC), return‐to‐work rate and satisfaction were not significantly lower for these patients [5].

Dejour et al. [9] and Metcalfe et al. [27] have found inferior clinical results in patients with a history of previous surgery. However, when analysing the current data, there was no statistically significant difference in outcome between patients with and without previous patella stabilising surgery (Table 3), even though patients with previous and postoperative additional surgery showed smaller increases in PROM scores than the other patients. The subgroups were small with limited power in the statistical analyses, and the differences could be clinically relevant, although not statistically significant (Table 3).

It is a limitation that this is an observational study following one specific treatment algorithm. Therefore, the results are not necessarily representative for other treatment strategies.

The strengths of the study are the structured approach with a fixed treatment algorithm, its prospective nature and the almost complete information on redislocation. As the treatment algorithm has been used for over 10 years with only a small alteration related to the diagnosis of rotational alignment and the study cohort covers 72% of all trochleoplasties performed nationwide during the study period, selection bias is minimal and the results are generally valid.

By providing detailed information on diagnostic criteria, patient selection, surgical technique and clinical outcome, the study provides valuable information for the on‐going discussion on how patients with patello‐femoral instability and high‐grade trochlea dysplasia are best treated.

## CONCLUSION

Treatment according to the described à la carte algorithm for patients with patellar instability and high‐grade trochlear dysplasia had a low redislocation rate and significant improvements in PROM scores with no deterioration after 5 years. Based on these results the described algorithm for treatment of patellar instability in patients with trochlea dysplasia seems useful in daily practice.

## AUTHOR CONTRIBUTIONS

Christian Dippmann conceived the study and wrote the manuscript together with Michael Rindom Krogsgaard with input from all authors. Michael Rindom Krogsgaard, Peter Lavard and Anette Holm Kourakis were in charge of patient recruiting, treatment and data registration. Christian Dippmann and Volkert Siersma extracted and analysed all clinical data. Philip Hansen and Monica Talibi assisted on radiological analysis and helped to establish interrater intraclass correlation.

## CONFLICT OF INTEREST STATEMENT

The authors declare no conflict of interest.

## ETHICS STATEMENT

Ethical approval was not needed for the present study. Formal consent was given by all participating patients.

## Supporting information

Supporting information.

Supporting information.

Supporting information.

## Data Availability

The data are not publicly available due to legal restrictions regarding the privacy of research participants.
