# Supplementary material for: Good 5‐year results and a low redislocation rate using an à la carte treatment algorithm for patellofemoral instability in patients with severe trochlea dysplasia
Source: Knee Surg Sports Traumatol Arthrosc. 2024 Aug 22;33(2):401–12. doi: 10.1002/ksa.12432 (PMC11792106; doi:10.1002/ksa.12432)
Supplement: Supplementary file 1 — Supporting information. [file KSA-33-401-s003.rtf]

Supplementary material 1:

Surgical technique: The knee is approached through a midline skin incision. The joint is opened through a lateral arthrotomy, dividing the vastus lateralis tendon. If a distalisation of the tibial tuberosity is planned, the osteotomy of the tibial tuberosity is performed first to secure easy access to the trochlea.
Using straight and curved osteotomes an osteochondral trochlea flap is raised, starting proximally and ending at the level of the intercondylar notch. The osteochondral flap is prepared with a thin layer of bone. A new trochlea is created in the bone, keeping as much height as possible on the lateral facet of the trochlea to normalize the inclination. The shape of the patellar joint surface is also considered when the trochlear groove is created to secure patello-femoral congruency. The cartilage flap is modelled into the groove and fixed centrally with a resorbable 10 mm Vicryl tape (Ethicon, Johnson & Johnson) through two drillholes to lateral femur and tied (fig. 2). The vastus lateralis tendon is sutured back in place, and if a tibial tuberosity transfer (medialisation and/or distalisation) is planned, this is carried out before performing the MPFL-R. The tibial tuberosity transfer is secured by two 4,5mm cannulated screws.
Finally, the MPFL-R is performed, using a gracilis autograft which is looped inside the proximal half of the patella through converging drillholes, passed just outside the medial knee capsule and inserted into a femoral drill tunnel at the Schoettle point identified by guidance of fluoroscopy.
Passive ROM is tested to ensure proper tension and isometry of the MPFL-R, and the graft is fixed to femur with a bio-interference screw. In rare cases with permanent patella dislocation a proximal release along the vastus lateralis muscle can be necessary. The lateral capsulotomy is left open while subcutis and the skin is closed separately. Cases of valgus malalignment are addressed with either a lateral open wedge distal femoral osteotomy or a medial closed wedge high tibial osteotomy, depending on the anatomical axis. In cases with rotational malalignment a distal femoral de-rotating osteotomy and/or a proximal high tibial osteotomy is performed [8, 40].

Post-operative regime:
Post-surgery, the knee is immobilized in a hinged brace at 30 degrees flexion for two weeks, allowing 50% weight bearing. Subsequently, range of motion (ROM) increases to 0-90 degrees over the next four weeks, with weight bearing as tolerated. Discharge occurs once patients can move comfortably, typically the day after surgery. Pain is managed with single-shot femoral nerve block. Thromboprophylaxis is given for the initial two first weeks using Tinzaparin (Innohep® 3500IE). Patients are referred to a 12-week supervised rehabilitation course free of charge.
The physiotherapist reports lack of expected progress to the surgeon, and if arthrofibrosis is suspected, early surgical intervention (< 8 weeks) is initiated. This involves arthroscopic adhesion removal and mobilization under general anaesthesia, followed by intense physiotherapy. Following a normal rehabilitation, a passive flexion of 120 degrees is expected at the three-months follow-up.  
