# Supplementary material for: Good 5‐year results and a low redislocation rate using an à la carte treatment algorithm for patellofemoral instability in patients with severe trochlea dysplasia
Source: Knee Surg Sports Traumatol Arthrosc. 2024 Aug 22;33(2):401–12. doi: 10.1002/ksa.12432 (PMC11792106; doi:10.1002/ksa.12432)
Supplement: Supplementary file 2 — Supporting information. [file KSA-33-401-s001.rtf]

Supplementary material 2:

Assessment of inter-rater inter-class correlation and limits of agreement in the measurement of lateral trochlea inclination (LTI) angle by three observers:

	ICC	LoA	
Before	0.647	[-8.00 ; 7.16]	
After	0.741	[-7.22 ; 5.98]	
Before + After	0.594	[-7.58 ; 6.51]	

Reliability is assessed by inter-rater intra-class correlation (ICC), measured by the fraction of residual variance in the total variance assessed in a linear mixed model including a random effect for rater. Agreement is assessed by 95% limits of agreement (LoA), calculated by the empirical 95% reference interval (2.5% and 97.5% percentiles) of the three sets of LTI differences between two raters. Both reliability and agreement was better for the measurements done at follow-up after the operation, than for the measurements before the operation, possibly because LTI<11 was a requirement for the operation. At the follow-up reliability was moderate, almost good, and the agreement was considered enough to assess clinical improvement, but not good enough to determine the LTI<11 threshold.   
