# Supplementary material for: Good 5‐year results and a low redislocation rate using an à la carte treatment algorithm for patellofemoral instability in patients with severe trochlea dysplasia
Source: Knee Surg Sports Traumatol Arthrosc. 2024 Aug 22;33(2):401–12. doi: 10.1002/ksa.12432 (PMC11792106; doi:10.1002/ksa.12432)
Supplement: Supplementary file 3 — Supporting information. [file KSA-33-401-s002.rtf]

Supplementary material 3:

Patient reported outcomes
When the study was planned, there was no valid PROM for patellar instability patients. The Kujala score [20] was widely used for these patients and therefore used in this study, but the items were developed without involvement of patients, meaning that it has no proven content validity, and the construct validity has never been tested using items response theory statistics [6]. It is a one-dimensional PROM with 13 items measuring from 0 (lowest knee functioning) to 100 (best knee functioning). Originally developed and validated in patients with anterior knee pain, the score is commonly used as well to evaluate the outcome after patella stabilisation surgery.

IKDC [17] was developed by experts without involvement of patient. It is meant to measure knee conditions in general, but it has no proven content validity. It contains 3 sections: Knee symptoms (7 items), function (2 items) and sports activities (2 items) but is reported as a single sum score ranging from 0 points (lowest level of function or highest level of symptoms) to 100 points (highest level of function and lowest level of symptoms). IKDC has not been validated for patients with patellofemoral instability

KOOS [23] contains three domains from the Western Ontario and McMaster Universities Osteoarthrtis Index (WOMAC) which was developed in 1986 for patients with end-stage osteoarthritis of hip or knee by involvement of this type of patients. Two domains (function in sport and recreation, and knee-related quality of life) were added in the mid-1990s by involvement of patients with knee problems, but not patellofemoral instability. Therefore, there is no certainty that it has content and construct validity for patients with patellofemoral instability.

The Lysholm [38] scale was developed for patients with knee (cruciate ligament) instability, but patients were not involved, and it has no proven content and construct validity for patients with patellofemoral instability. The total score is the sum of all eight questions and can range from 0-100. Higher scores indicate better clinical status/outcome. Scores are commonly categorized as excellent (95-100), good (84-94), fair (65-83) and poor (<65).

There is no estimated minimal clinical important difference for scores obtained by these PROMs in patients with patellofemoral instability. However, a difference of 50% of the preoperative standard deviation (SD) between preoperative and postoperative scores for a given PROM is a commonly used threshold for important patient-perceived change [23].
